# Bisphenol A Exposure *in utero* Disrupts Hypothalamic Gene Expression Particularly Genes Suspected in Autism Spectrum Disorders and Neuron and Hormone Signaling

**DOI:** 10.3390/ijms21093129

**Published:** 2020-04-29

**Authors:** Anne D. Henriksen, Alejandro Andrade, Erin P. Harris, Emilie F. Rissman, Jennifer T. Wolstenholme

**Affiliations:** 1 Department of Integrated Science and Technology, James Madison University, Harrisonburg, VA 22807, USA; henrikad@jmu.edu; 2 Department of Pharmacology and Toxicology, Virginia Commonwealth University, Richmond, VA 23298-0613, USA; andradea@mymail.vcu.edu; 3 Center for Human Health and the Environment, North Carolina State University, Raleigh, NC 27695, USA; eph4tk@virginia.edu (E.P.H.); efrissma@ncsu.edu (E.F.R.)

**Keywords:** endocrine disruptor, bisphenol A, neurodevelopment, genomics, syntaxin 1a

## Abstract

Bisphenol A (BPA) is an endocrine-disrupting compound detected in the urine of more than 92% of humans, easily crosses the placental barrier, and has been shown to influence gene expression during fetal brain development. The purpose of this study was to investigate the effect of in utero BPA exposure on gene expression in the anterior hypothalamus, the basal nucleus of the stria terminalis (BNST), and hippocampus in C57BL/6 mice. Mice were exposed in utero to human-relevant doses of BPA, and then RNA sequencing was performed on male PND 28 tissue from whole hypothalamus (*n* = 3/group) that included the medial preoptic area (mPOA) and BNST to determine whether any genes were differentially expressed between BPA-exposed and control mice. A subset of genes was selected for further study using RT-qPCR on adult tissue from hippocampus to determine whether any differentially expressed genes (DEGs) persisted into adulthood. Two different RNA-Seq workflows indicated a total of 259 genes that were differentially expressed between BPA-exposed and control mice. Gene ontology analysis indicated that those DEGs were overrepresented in categories relating to mating, cell–cell signaling, behavior, neurodevelopment, neurogenesis, synapse formation, cognition, learning behaviors, hormone activity, and signaling receptor activity, among others. Ingenuity Pathway Analysis was used to interrogate novel gene networks and upstream regulators, indicating the top five upstream regulators as huntingtin, beta-estradiol, alpha-synuclein, *Creb1*, and estrogen receptor (ER)-alpha. In addition, 15 DE genes were identified that are suspected in autism spectrum disorders.

## 1. Introduction

Bisphenol A (BPA) is an endocrine-disrupting compound used in the manufacture of everyday products such as plastic water bottles, canned food linings and thermal receipts. Due to its ability to leach from these products, BPA is found ubiquitously in the environment and has been detected in the urine of more than 92% of people tested [1]. BPA acts at several steroid receptors and has weak estrogenic activity at the estrogen receptor (ER)-alpha, ER-beta, and G-protein-coupled ER-1 (GPER1). It also antagonizes the androgen receptor and blocks aromatase enzyme activity [2]. BPA modulates thyroid receptors and can act via non-genomic, intracellular signaling by binding membrane ERs such as GPER1 [3]. Thus, BPA can disrupt endocrine signaling—important for many biological functions such as growth, metabolism and reproduction—and can impact social behaviors.

The developing fetus is especially vulnerable to such endocrine disruption, as the brain is in a state of rapid growth, including neurogenesis, differentiation, and migration over a relatively short period of time. Since sex steroid hormones guide brain development from gestation through the neonatal period, gestational exposure to BPA may disturb this development and lead to disruption in the mature brain. BPA exposure alters neuroendocrine control of hormonal signaling and neurotransmitter control of nervous system function, and can lead to lasting changes in a number of behaviors including reproductive, emotional, learning, memory, and social [4,5]. Human neurodevelopmental studies have shown that early-life exposure to BPA is associated with increased externalizing behavior in two-year-old girls [6], increased anxiety and depression in three-year-old girls [7], increased attention/hyperactivity in four-year-old boys and girls [8], and increased aggression in school-aged boys [9,10]. Rodent and model organism studies have reported similar effects in young animals following early-life BPA exposure. Rodent studies have documented hyperlocomotor activity, anxiety-like, and depressive-like behaviors [11,12,13,14,15]. Social and cognitive deficits have also been found; however, the extent of these changes appears to depend on the timing of exposure, the timing of the test, and the nature of the phenotypes measured [3]. Some of these alterations can be passed on to future, unexposed, offspring from a single generational BPA exposure [16,17,18].

The hypothalamus and the hippocampus appear to be areas of particular vulnerability to BPA’s action [3]. The hypothalamus responds to endocrine-disrupting chemicals and is one of the primary brain areas governing many sociosexual behaviors. Thus, we hypothesized that gestational exposure to BPA would induce global transcriptomic changes in the hypothalamus and that some of these changes would persist into adulthood. We used RNA-Seq to determine the global transcriptomic alterations in the hypothalamus of PND 28 juvenile male C57BL/6J mice exposed to BPA throughout gestation. This juvenile period was chosen as it reflects the hypothalamic gene expression patterns altered in adolescence, a period in which we and others have noted BPA-induced changes in social and memory-related deficits [2,12,14,16,19]. We then tested putative signaling pathways to determine whether any were persistently altered in adults following gestational BPA exposure. These studies were designed to investigate gene expression changes in the hypothalamus following gestational exposure to low-dose BPA. Since many of these gene expression changes were found to be overrepresented in categories relating to neurodevelopment, neurogenesis, synapse formation, and learning behaviors, we extended these findings to determine whether candidate gene expression effects persisted into adulthood in a brain region, the hippocampus, known for playing a role in learning and memory.

## 2. Results

### 2.1. RNA-Sequencing and Analysis

In Experiment 1, tissue from male PND 28 offspring of dams exposed to human-relevant levels of BPA during gestation was used for RNA-sequencing analysis. Total RNA from the bed nucleus of the stria terminalis and the medial preoptic area of the anterior hypothalamus (BNST/mPOA-HT) was analyzed for gene-level expression differences using two independent analysis workflows to interrogate differential gene expression between the BPA-exposed males and controls: STAR/featureCounts/DESeq2 and Galaxy TopHat2/Cuffdiff. Using an absolute value of the log fold change cutoff >0.05 and an adjusted *p* value <0.05, the STAR/featureCounts/DESeq2 analysis indicated 77 genes that were differentially expressed between BPA and control mice. The Galaxy TopHat2/Cuffdiff analysis identified 245 genes that were differentially expressed between BPA and control mice, using the same fold change and significance cutoffs. Of these, 63 genes were common to both analyses (Table 1 and Supplemental Table S1, genes in bold) and of those genes, 27 were upregulated by BPA and 36 were downregulated by BPA.

**Figure 1:**
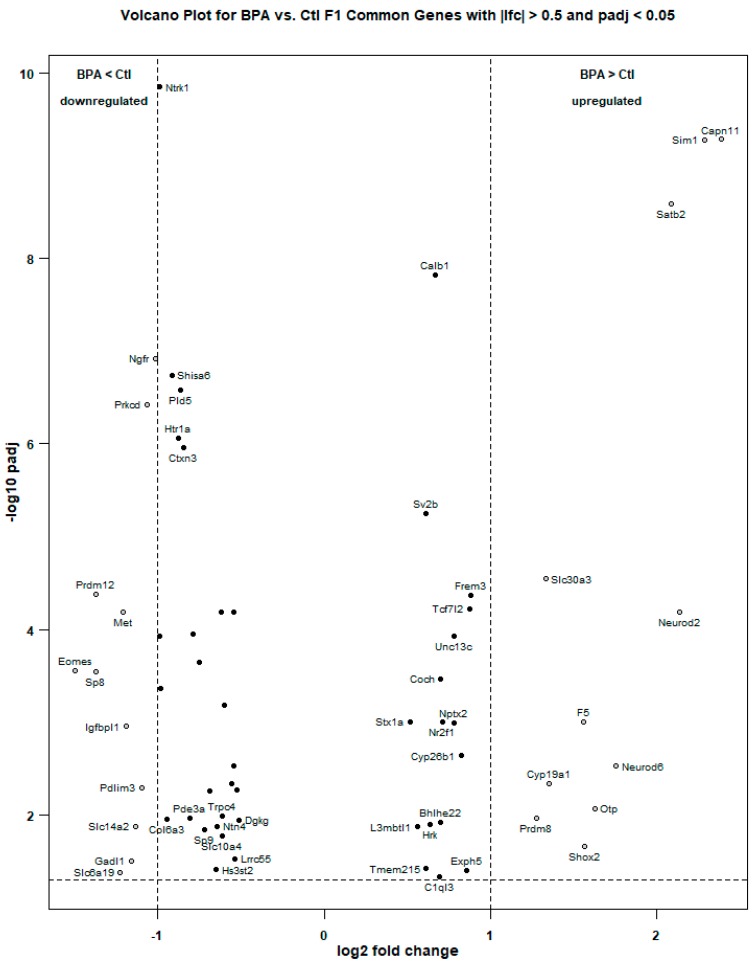
Volcano plot of the 63 differentially expressed genes in the F1 generation common to the STAR/featureCounts/DESeq2 and TopHat/Cuffdiff RNA-Seq analyses. Only the section of the log-scale y axis for which the adjusted *p* value is less than 0.05 (−log_10_(0.05) = 1.3) is shown. The higher up and further to the edges that a gene is situated, the greater the effect size (higher log fold change) and the higher the statistical significance (lower adjusted *p* value) of the differential expression. Four genes were determined to be upregulated by a fold change of more than 2 and 11 genes were determined to be upregulated by a fold change of more than 1 (dots on the right) under direct exposure in utero to bisphenol A (BPA). Eleven genes (dots on the left) were determined to be downregulated by a fold change of more than 1 under direct exposure in utero to BPA.

**Figure 2:**
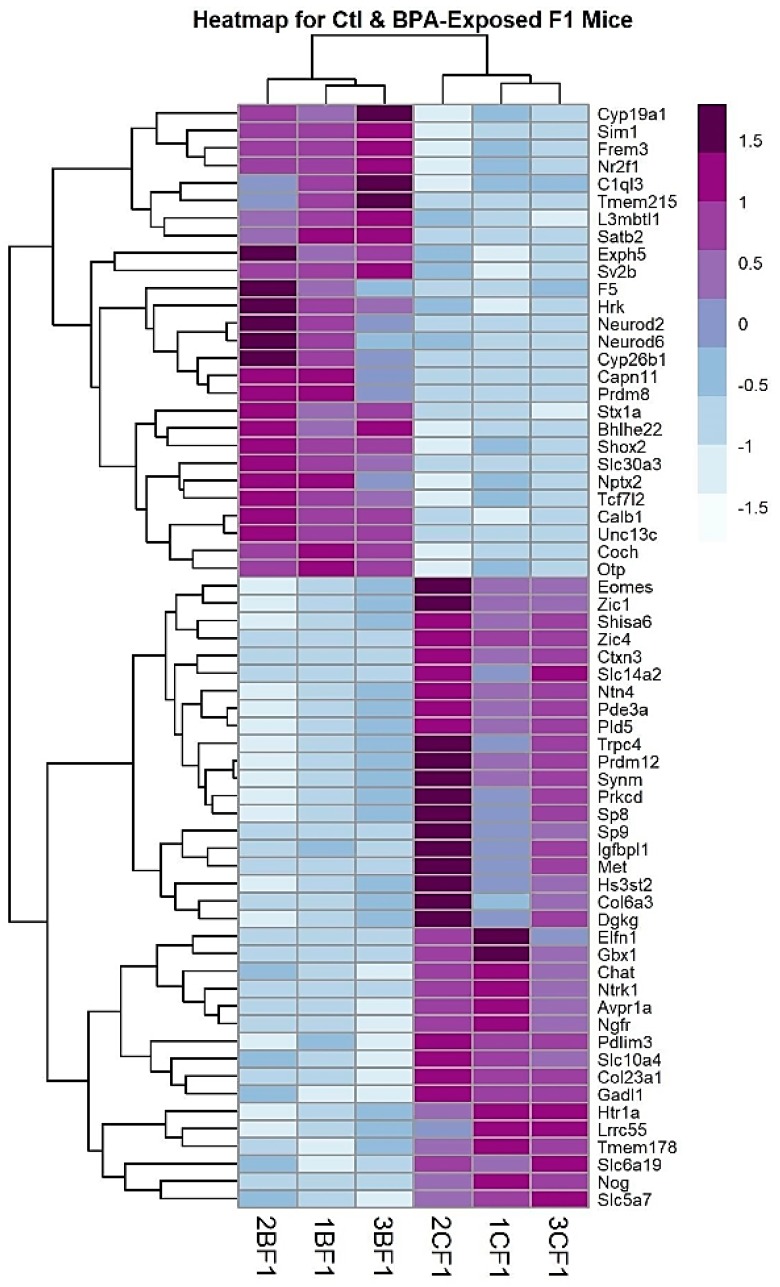
Heatmap and clustering diagram for the 63 differentially expressed genes in the F1 generation common to the STAR/featureCounts/DESeq2 and TopHat/Cuffdiff RNA-Seq analyses with adjusted *p* values < 0.05 and log fold change absolute values >0.5. The clustering and heatmap were done in R Statistical Programming Language with the pheatmap package using correlation distance and average linkage. The color of the cells indicates the standardized number of normalized mRNA reads of the F1 BPA and control samples for that gene (upregulated = purple; down regulated = blue). Note that the biological replicates for BPA and control cluster together.

Following differential expression analysis, the RNA-Seq results were visualized multiple ways to highlight significant genes. The abbreviated volcano plot in Figure 1 shows the 63 most statistically significant, differentially expressed, genes common to both RNA-Seq workflows. To visualize genes whose expression levels may be similarly regulated following gestational BPA exposure, genes were hierarchically clustered based on a mathematical algorithm using the Pearson correlation distance and average linkage on scaled rows. The Pearson correlation is a measure of the difference between the expression profiles across samples of two genes using the equation for the Pearson correlation coefficient between the gene expression vectors. This measure of “distance” causes genes with similar expression profiles (i.e., shorter distance) to group together in the heatmap. Unlike Euclidean distance, the Pearson correlation is relatively insensitive to outliers and scaling. The distance between genes (or gene clusters) was calculated using the average linkage between clusters; that is, the average of the distance of each point in one cluster to every point in the other cluster. This method groups genes with similar expression profiles across biological replicates. In cluster diagrams, genes whose expression levels are closely regulated across replicates will cluster together and may be involved in related biological pathways. Clustered genes are depicted in a heatmap (Figure 2), where colors indicate relative expression levels of a gene across experiments. Progressively similar gene expression profiles are then organized in a hierarchical tree structure dendrogram. We show the 63 genes common to both analyses that are differentially expressed between BPA-exposed and control mouse brain in the F1 generation.

### 2.2. Genes Involved in Cell Signaling, Hormone Activity, and Learning Behavior are Overrepresented in BPA Males

To increase identification of common sets of genes or signaling pathways altered by gestational BPA exposure, we used the union of our analysis pipelines in our subsequent bioinformatics analyses. We combined the two sets of the significantly altered, differentially expressed genes from the STAR/featureCounts/DESeq2 analysis (*n* = 245) and Galaxy TopHat2/Cuffdiff (*n* = 77) analysis, resulting in a total of 259 genes (Supplemental Table S1). Gene Ontology overrepresentation analysis identified multiple categories involved in cell–cell signaling; behaviors related to cognition, learning and memory; and mating (Supplemental Table S2). We used Revigo to reduce the complexity and redundancy of each of the GO categories using semantic language processing (Supplemental Figure S1). The top significant categories that were overrepresented in our dataset for Biological Processes included cell–cell signaling, behavior (specifically cognition, learning and memory, and mating), response to endogenous stimuli, and metal ion transport. The top Cellular Component categories were similar in response and were enriched for neuron projection, synapse, and glutamatergic synapse. In Molecular Functions, the top categories included RNA polymerase II-specific transcriptional activator activity, calcium-independent PKC activity, hormone activity, and extracellular matrix structural constituent and signaling receptor activity.

We also used the Ingenuity Pathway Analysis (IPA, www.ingenuity.com) to interrogate novel gene networks and potential upstream regulators. One advantage of IPA is that it can identify upstream regulators that may help explain the genes that are differentially regulated in BPA-exposed adults. Intriguingly, the five top upstream regulators were huntingtin, beta-estradiol, alpha-synuclein, Creb1 and ER-alpha (Supplemental Figure S2). IPA revealed many overrepresented canonical pathways in common with our Gene Ontology analysis. For example, G-protein-coupled receptor signaling, CREB signaling, and GnRH signaling were overrepresented by IPA, while our Gene Ontology analysis found hormone activity, chromatin binding, and signaling receptor activity as overrepresented. Of particular interest in the IPA analysis, learning, cognition, motor dysfunction, neurological disease and abnormal physiology of the nervous system were the top diseases and functions significantly overrepresented in our gene set.

### 2.3. Comparison with Other Published Genomic Datasets

Only a handful of studies have published genomic expression changes following pre- or post-natal BPA exposure in rodent brain tissue. Although the studies differed in windows of exposure, BPA concentrations, methods of administration, and species of rodent, we compared our hypothalamic DEGs (*n* = 259) with those derived from one *Mus musculus* [20], one *Peromyscus californicus* [21] and one Sprague Dawley rat study all using hypothalamus tissue [22]. As part of a larger -omics study on cardiometabolic disorders, C57BL/6J male mice were dosed with 5mg/kg BPA by daily gavage from gestational day 0 (GD 0) to birth (PND 0) [20]. Using whole hypothalamus collected from males at PND21, Shu et al. identified 994 genes (at *p* < 0.05 via RNA-Seq analysis) that were differentially expressed in BPA-exposed hypothalamus compared to vehicle controls. Sprague Dawley rats from the CLARITY study were dosed with 2.5 or 2500 μg/kg BPA daily from GD 6 to birth, and whole hypothalamus was collected at PND 1 and used for RNA-Seq analysis [22]. Genes that were differentially expressed at padj <0.05 in both doses as compared to vehicle (*n* = 371 DEGs) were compared to our analysis. In Johnson et al. 2017, *P. californicus* dams were placed on a diet enriched in BPA (50 mg/kg feed weight) through gestation and lactation. Male and female whole hypothalamus was collected at weaning, used for RNA-Seq analysis, and 557 genes combined from separate male and female analyses were differentially expressed at FDR <0.05 [21]. Likely due to the differences in rodents, doses, windows of exposure, and age of tissue assayed, we found only 18 genes in common (Supplemental Figure S3) with the other C57BL/6J study [20] and 6 genes in common with the *P. californicus* offspring [21]. No genes were in common with the CLARITY rat study [22]. Of note, the genes that were in common between our analysis and publicly available data were enriched for RNA polymerase-specific DNA binding, neuron projections, and serotonergic synapse.

Given the putative role of endocrine-disrupting compounds (EDCs) in autism spectrum disorders (ASD) [23,24,25] and several reports of disrupted social behavior following pre- or peri-natal BPA exposure, we also interrogated two publicly available databases of genes curated from humans and animal models that are suspected to act in ASD: SFARI gene and AutismKB 2.0. These datasets are manually curated from the published literature for autism-risk genes. Using the SFARI gene February 2020 freeze (https://gene.sfari.org/), 913 genes were identified in the human gene database and these comprise the most comprehensive, up-to-date reference for all known human genes associated with autism spectrum disorders. We found 15 genes in common with the SFARI human gene list. As expected, the vasopressin 1a receptor (*Avpr1a*) and oxytocin (*Oxt*) were in both datasets. Notably, a few genes involved in learning and memory—*Camk4*, *Satb2* and *Stx1a*—were also present in both datasets. The AutismKB 2.0 database consists of 229 (30 syndromic and 198 non-syndromic) autism-related genes (http://db.cbi.pku.edu.cn/autismkb_v2/index.php). Seven genes were in common between the syndromic and non-syndromic autism-related genes and our dataset and included *Avpr1a* and the glutamate transporter (*Slc6a13*). In general, the lack of a robust overlap between published data and our dataset (Supplemental Figure S3) could suggest one of two things. First, BPA may play a minor role in altering the genome in a similar manner as ASD, or second, BPA and ASD phenotypes are unrelated, but may share a common pathway to disrupt social learning phenotypes.

### 2.4. Adult Hippocampal Expression of Genes Involved in Learning and Memory

**Figure 3:**
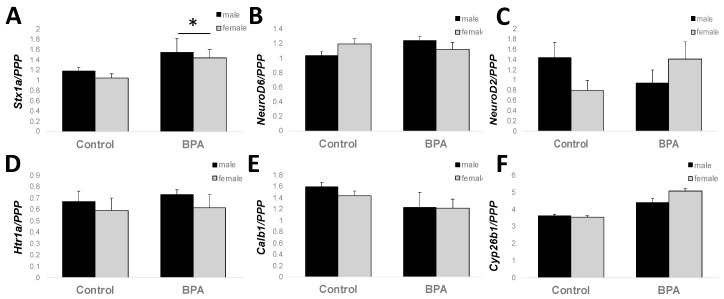
Gene expression changes in adult hippocampus. Genes involved in learning were assayed by qPCR. (**A**) *Stx1a* showed a main effect of treatment. (**B**) *NeuroD2* and (**C**) *NeuroD6* showed a trend for an interaction between sex and BPA treatment. (**D**) *Htr1a*, € *Calb1*, and (**F**) *Cyp26b1* did not show an effect of treatment or sex. Data are reported as the mean ± SEM. * *p* < 0.05.

**Figure 4:**
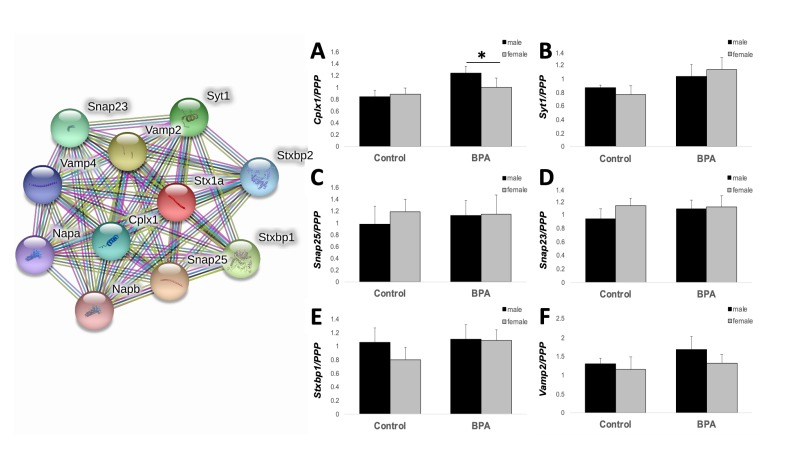
Gene expression changes in adult hippocampus. STRING protein–protein interaction network for Stx1a. Genes with known *Stx1a* interaction at the synapse were assayed by qPCR. (**A**) *Cplx1* showed a main effect of treatment. (**B**) *Sytt1* showed a trend for an increase with BPA treatment. (**C**) *Snap25* (**D**) *Snap23*, (**E**) *Stxbp1*, and (**F**) *Vamp2* did not show an effect of treatment or sex. Data are reported as the mean ± SEM. * *p* < 0.05.

Because all tissues from adult hypothalamus were used for other studies by the time we completed these analyses, we extended our findings from juvenile male hypothalamus to adult male and female hippocampus. The hippocampus was selected as a region of interest because it is critically involved in learning and memory and is sensitive to stress [26]. Several genes overrepresented in our Ingenuity Pathway Analysis and Gene Ontology analysis in juvenile males exposed to gestational BPA are involved in cognition, learning and memory (Figure 3): *NeuroD2*, *NeuroD6*, *Calb1*, *Htr1a*, *Cyp26b1*, and *Stx1a.* Of the genes tested, two-way ANOVA revealed a main effect of BPA treatment on *Stx1a* expression, where BPA increased expression in both males and females (F_1,23_ = 5.330, *p* = 0.034). *NeuroD2* and *NeuroD6* showed a trend for an interaction between treatment and sex (F_1,23_ = 4.128, *p* = 0.056 and F_1,23_ = 4.234, *p* = 0.053, respectively). Given the known role of *Stx1a* in hormone and neurotransmitter exocytosis, and the importance of protein–protein interactions when involved in docking of synaptic vesicles in pre-synaptic active zones and our previous study on the transgenerational effects of BPA on gene expression in the post-synaptic density, we used the STRING protein–protein interaction database (https://string-db.org/) to interrogate other genes known to interact with *Stx1a* at the synapse. Ten proteins were identified to directly interact with STX1A (Figure 4). We designed primers for six of these genes known to play a role in the docking and fusion of synaptic vesicles: *Snap25*, *Vamp2*, *Stxbp1*, *Syt1*, *Snap23*, and *Cplx1*. Similar to *Stx1a*, *Cplx1* showed a main effect of BPA treatment (F_1,23_ = 4.436, *p* = 0.048), while *Syt1* showed a trend for an increase in BPA-exposed male and female mice (F_1,23_ = 3.619, *p* = 0.072). No other gene was significantly altered by sex or BPA exposure.

## 3. Discussion

The aim of these studies was to identify gene expression changes in juvenile hypothalamus tissue following gestational exposure to human-relevant levels of BPA. We chose this age because BPA alters juvenile social behavior [2,17,18], cognition, and spine morphology [27,28,29,30]. These differential gene expression differences can help identify mechanisms underlying actions of gestational BPA exposure on behavior. We report modest differential gene expression in juvenile hypothalamus; only ~250 genes passed our fold change and adjusted *p* value cutoffs. We used two RNA-Seq analysis methods with differing methods for calling differentially expressed genes. DESeq2 accounts for high dispersion across small replicate numbers with low reads and displays the “shrunken” lfc value, resulting in a more conservative selection of 77 DEGs. The Galaxy Cuffdiff workflow generates the lfc of the fragments per kilobase of transcript per million mapped reads (FPKM) and identified 245 DEGs. We chose to use the union of the two analysis methods (*n* = 259 DEGs) for our bioinformatics analysis to increase our ability of identifying common signaling pathways. Indeed, these genes showed a remarkably cohesive enrichment for cell signaling, learning and memory, and the synapse. Genes involved in hormone response and signaling were also enriched and estrogen was identified as a top regulator of these BPA-induced DEGs. To determine whether these changes persisted into adulthood, we investigated the expression of genes involved in learning and memory in adult hippocampal tissue. We focused on one gene, syntaxin 1a, *Stx1a*, as a long-term target for BPA disruption due to its known role in the regulation of hormone and neurotransmitter release at the synapse. *Sxt1a* expression, and one of its binding partners, complexin 1 (*Cplx1*), were increased in the hippocampus of adults exposed to gestational BPA, suggesting a long-term disruption of synaptic plasticity.

Our genomic survey of hypothalamic gene expression following gestational BPA exposure revealed two main overrepresented categories: genes involved in hormone signaling and genes involved in cell–cell signaling, learning, cognition, and the synapse. Some of the expected estrogen-related genes were altered by BPA in our dataset. For example, estrogen receptor alpha (*Esr1*), estrogen-related receptor gamma (*Esrrg*), arginine vasopressin (*Avp*), and aromatase (*Cyp19a1*) were significantly increased in juvenile BPA-exposed male hypothalamus, while *Avpr1a* expression was decreased. Additionally, the top upstream regulators analysis identified β-estradiol and ER-alpha as two of the five top upstream regulators of the differential gene expression changes in the hypothalamus of juveniles exposed to gestational BPA. In a previous study from our laboratory, we noted that a similar BPA exposure paradigm reduced *Avp* and *Esr1* mRNA in whole embryo brains [17], and decreased ER-alpha immunoreactivity in specific regions of the hypothalamus in BPA-exposed males as compared with control males [31]. One reason for our modest but opposite response in estrogen receptor expression differences in this study could be due to the fact that we surveyed the whole hypothalamus rather than its individual subregions. In fact, the study using immunoreactivity is proof of principle that these effects are tightly regulated in specific nuclei. Thus, region-specific differences in ER expression (between sexes and/or exposure groups) are homogenized and, consequently, not fully detectable in whole hypothalamus. Additionally, these studies were performed in adult tissue, and the differences may reflect developmental influences on *Esr1* and *Avp*.

Although our gene set only minimally overlapped with other published datasets on rodents with similar BPA exposure, we found similar enriched biological pathways were in common between the studies. The CLARITY study investigating gene expression differences in Sprague Dawley rats found enrichment of regulation of transcription, nervous system development and function, and morphology of dendritic spines [22]. Likewise, disruption of spine morphology has previously been associated with BPA exposure in rodents and non-human primates of both sexes [27,28,29]. The only GO term enriched in the *P. californicus* study was microtubule-based processes [21], and relatedly, the only enriched term in the hypothalamus dataset from the cardiometabolomic study was extracellular matrix-related processes [20]. In an RNA-Seq analysis of hippocampal tissues isolated from male and female neonatal rats exposed to 5 mg/kg BPA in utero, many of the same canonical pathways were similar to our findings, despite different brain regions being sampled. In males, top canonical pathways (glutamate receptor signaling, axonal guidance signaling, and circadian rhythm signaling) and several neurological functions (morphogenesis of neurons, neuritogenesis, and formation of brain) were significantly associated with in utero BPA [32].

Growing lines of convergent evidence suggest that BPA may have lasting effects, even transgenerational effects on brain development, specifically on cell–cell communication and synaptic plasticity. Direct exposure to BPA alters synaptic spine morphology [27,28,29,30] and synaptic plasticity [33]. BPA exposure from gestation through lactation thickened post-synaptic densities, and increased curvature of synapses in hippocampal pyramidal cells [34]. Peri-natal [34,35] or gestational BPA exposure [16] reduced several PSD-related proteins. Neurogenesis and neuronal migration can be accelerated [36,37] or delayed [14,38] in rodents exposed to BPA during gestation. Due to the fact that learning and memory, cognition, glutamatergic synapse, and cell–cell signaling were all significantly overrepresented in our gene set, and the fact that we have previously found post-synaptic density proteins and synaptic plasticity genes, specifically Shank1 and mGlur5, were decreased in juvenile brains three generations after gestational BPA exposure, we surveyed expression of genes involved in learning in adult male and female hippocampus. *Stx1a* showed significantly increased expression in both males and females, whereas *NeuroD2* and *NeuroD6* showed a trend for a significant interaction between BPA exposure and sex. *NeuroD6* and *NeuroD2* play a role in neuronal differentiation and *NeuroD2* may play a role in emotional learning [39]. Of interest, *Stx1a* may be specifically involved in hormone and neurotransmitter release. Knockout (KO) mice without *Stx1a* reveal its role in learning, memory, and synaptic plasticity. For example, *Stx1a* KO mice have impaired long-term potentiation, consolidation of conditioned fear memory, and impaired conditioned fear memory extinction, although they exhibit normal spatial memory [40]. Importantly, these effects appear to be limited to STX1A dysfunction since the viable *Stx1a* KO did not alter expression of other SNARE proteins [40]. This mimics our gene expression results; many of the known STX1A binding proteins did not significantly differ in their expression of *Stxbp1*, *Vamp2*, *Syt1*, *Snap23*, or *Snap25*. The one gene that was significantly decreased, *Cplx1*, is involved in the late stages of vesicle exocytosis and may be a downstream effect of *Stx1a* dysregulation.

In alignment with our previous findings on BPA-induced social interaction deficits [18], *Sxt1a* KOs and heterozygotes (+/-) also display abnormal behavior in social interaction and novel object exploration [41,42]. STX1A misregulation may play a role in a subpopulation of autistic patients, as a small study has observed that some ASD patients had haploidy of STX1A gene and lower STX1A gene expression [43]. Behaviorally, we have consistently found social recognition deficits, while our differential gene expression results add to the growing studies suggesting that BPA interferes with synaptic plasticity. This may be a mechanism through which BPA disrupts proper migration of cells/proper formation of the synapse and leads to deficits in a social memory task, and these effects may last into future generations. We have previously reported direct and transgenerational actions of BPA on genes and proteins involved in estrogen signaling [2,17,31] and in the post-synaptic density [16]. Here, we suggest BPA may also be acting pre-synaptically as well. Overall, our findings add to the growing literature suggesting a common mechanism for BPA misregulation of cell–cell communication, and this may lead to associated social and cognitive behavioral impairments.

## 4. Materials and Methods

### 4.1. Animals and Tissue Collection

C57BL/6J mice were purchased from Jackson Laboratories (Bar Harbor, ME, USA country) and housed at the University of Virginia or North Carolina State University (NCSU). The animals used in this research were housed at the Biological Resources Facility at NCSU. The animal protocol (14-159-B) was approved first on 2/2015 and renewed yearly thereafter by the Institutional Animal Care and Use Committee at North Carolina State University. Mice were maintained on a 12:12 light:dark cycle. Adult females were randomly assigned to a control phytoestrogen-free diet (Harlan Teklad TD95092), or to the same diet with 5 mg BPA/kg diet added (Harlan Teklad TD09386). In a prior study using the same BPA exposure paradigm, the BPA concentrations in dam serum were equivalent to those found in humans [2]. As previously described [2,17,18], females were placed on their assigned diets 7–10 days before pairing with a male. After one week, males were removed, and females continued consuming their assigned diets ad libitum. Within twelve hours of birth, four littermates from BPA or control dams were fostered to dams consuming control diet who had given birth within the previous 24 h. Foster dams retained two of their own pups; these mice were not used in our studies. Thus, BPA exposure in these studies was restricted to pre-mating and gestation. At weaning, on post-natal day 21 (PND 21), mice were placed on standard chow (Harlan Teklad Diet #7912, which contains phytoestrogens), and were group housed (3–5 per cage) by litter and sex.

In Experiment 1, mice were harvested on PND 28, and BNST/mPOA-hypothalamus tissue was collected in a single free-hand dissection, as previously described [44]. Total RNA was isolated from F1 BPA males (*n* = 3) and control males (*n* = 3) and sent to Expression Analysis (EA, Durham, NC, USA) for sequencing. Each male sampled was from a different litter. Poly-A tailed mRNA and lncRNA were extracted by EA and quality tested. EA synthesized cDNA from the purified RNA and then amplified and sequenced the cDNA on an Illumina Hi-Seq 2000. Between 30.7 and 46.7 million, fifty base-pair, paired-end reads were obtained for each of the six samples. The fragment size was 175 base pairs, leaving 75 bases between the 50-base-paired-end reads unsequenced. We restricted this analysis to males because we wanted to include genes from both sex chromosomes. In Experiment 2, adult male and female F1 mice (*n* = 6/group) were harvested on (PND 60–80); whole brain was frozen on dry ice and stored until processing. Brains were cut on a cryostat and hippocampal tissue was punched for RNA isolation and qPCR analysis.

### 4.2. RNA-Sequencing Analysis and Bioinformatics

Differential expression (DE) analysis was performed using two separate workflows to determine which genes were differentially expressed between BPA-exposed and control mice, as previously described [44]. Results from the two analysis methods were combined to generate a rigorously tested set of genes for downstream analysis. Briefly, the initial inspection and quality trimming of the FASTQ files was performed by Expression Analysis using ea-utils, a proprietary tool of that company. In our initial workflow, STAR was used to align the sequences to the mouse genome (mm9 [45]. STAR is an ultrafast, universal read alignment tool that is able to identify and map reads to splice junction sites. FeatureCounts was used to assign the aligned sequence reads to genomic features and to quantify the reads [46]. Differential expression analysis of the featureCounts raw counts matrix was conducted using DESeq2, a package in Bioconductor in the R Statistical Programming language [47]. DESeq2 accounts for high dispersion across small replicate numbers with low reads and displays the “shrunken” log fold change (lfc) value. Contrasts between BPA and control mice produced a log fold change (lfc) measure of effect size and an adjusted-*p* value measure of statistical significance for multiple comparisons for ~19,000 genes. The DESeq2-adjusted *p* value uses the Benjamini–Hochberg FDR correction for multiple tests. Genes were ranked by both lfc and adjusted *p* value to determine ones that were statistically significant between two conditions. In this experiment, genes with lfc absolute values greater than 0.5 and an adjusted *p* value of less than 0.05 were considered differentially expressed.

The analogous workflow was executed on the FASTQ files from within the Galaxy platform [48] using software that is part of the Tuxedo Suite for RNA-Seq analysis [49], as previously described [44]. The FASTQ files were first inspected for quality using FASTQC, and then trimmed by one base using FASTX Trimmer [50,51]. Next, alignment to the UCSC mm9 mouse reference genome was performed using TopHat2 [52]. TopHat2 alignment produced binary alignment map (BAM) files, which were then input to the Tuxedo Suite differential expression analysis tool, Cuffdiff [53]. The Cuffdiff workflow outputs the lfc of the fragments per kilobase of transcript per million mapped reads (FPKM) for ~19,000 genes with measurable expression levels, including effect sizes and adjusted *p* values, as well as other information on transcription start sites, coding sequences, promoters, and transcript-level differential expression. Cuffidff q values for each gene are reported as the Storey minimum unbiased FDR estimate for that gene.

Bioinformatic analysis was performed using previously established pipelines from the Wolstenholme and Rissman labs [44,54,55,56] and included functional overrepresentation analysis with Gene Ontology using the ToppFun suite of tools [57] in the ToppGene Suite for gene list enrichment analysis. Candidate gene prioritization and gene network mapping was performed using Ingenuity Pathway Analysis (www.ingenuity.com) to assess the biological significance and relationships between these genes, based on current scientific literature.

### 4.3. RNA Isolation and qPCR Analysis

Total RNA from hippocampus of adult male and female mice (*n* = 6/group) was isolated using STAT 60 reagent (Tel-Test, Friendswood, TX, USA) and RNeasy mini kit (Qiagen, Valencia, CA, USA) according to the manufacturer’s protocol. RNA concentration was determined by absorbance at 260 nm using a spectrophotometer (Nanodrop 2000, ThermoFisher Scientific, Madison, WI, USA). Total RNA was reverse transcribed into cDNA using the iScript cDNA synthesis kit (BioRad, Hercules, CA, USA). Real-time qPCR was performed on the CFX thermocycler (BioRad, Hercules, CA, USA) using the SsoAdvanced Universal SYBR Green Supermix (BioRad, Hercules, CA, USA) using standard protocols [17,54,55,56]. Biological replicate samples were run in triplicate. Quantification of candidate gene expression levels was calculated based on the threshold cycle (C_t_) for each well using the provided software and normalized to *PPP2r2p* as endogenous control. Relative changes in gene expression were normalized to the control male group. To extend the findings from our RNA-Seq analysis, we designed primers for genes in categories overrepresented in our Gene Ontology analysis. Primer sequences are listed in Table 2.

### 4.4. Statistical Analysis

Statistically significant differential expression for the RNA-sequencing dataset was defined in this study as a log fold change absolute value greater than 0.5 and multiple test-adjusted *p* value less than 0.05. The STAR/featureCounts/DESeq2 DE output and the Galaxy TopHat2/Cuffdiff DE output were examined to determine which genes fell into this category. The genes that were common to both approaches (*n* = 63 DEGs) were subjected to further exploratory data analysis and visualization, including volcano plots and heatmap clustering. The volcano plot and heatmap clustering were performed in R Statistical Programming Language. The union of the DEGs from both analyses (*n* = 259 DEGs) was used for Gene Ontology overrepresentation analyses and Ingenuity Pathway Analysis.

Gene expression data were analyzed using the SigmaPlot software, version 14 (Systat Software, Inc., San Jose, CA, USA) and are expressed as the mean ± SEM. Quantitative RT-qPCR expression data were analyzed using two-way ANOVA with sex and treatment as factors. Student–Newman–Keuls post-hoc tests were performed for alpha <0.05. Quality control statistics and bioinformatics used for genomic expression data are described above.

## Figures and Tables

**Table 1:** DEGs in common to two RNA-Seq workflows for in utero BPA-exposed F1 C57BL/6JJ mice.

DESeq2/Galaxy Union Genes			DESeq2 DE Results			Galaxy CuffDiff DE Results		
Gene	RefSeqID	Chr	lfc	*p* value	padj	log2fc	*p*_value	*q*_value
*Eomes*	NM_010136	9	−1.4979	0.0000	0.0003	−1.9697	0.0001	0.0055
*Prdm12*	NM_001123362	2	−1.3741	0.0000	0.0000	−1.4659	0.0001	0.0055
*Sp8*	NM_177082	12	−1.3726	0.0000	0.0003	−1.5349	0.0001	0.0055
*Slc6a19*	NM_028878	13	−1.2254	0.0003	0.0419	−1.9559	0.0001	0.0055
*Met*	NM_008591	6	−1.2116	0.0000	0.0001	−1.3163	0.0001	0.0055
*Igfbpl1*	NM_018741	4	−1.1905	0.0000	0.0011	−1.2246	0.0001	0.0055
*Gadl1*	NM_028638	9	−1.1615	0.0002	0.0313	−1.2434	0.0001	0.0055
*Slc14a2*	NM_001110274	18	−1.1338	0.0001	0.0133	−1.4175	0.0001	0.0055
*Pdlim3*	NM_016798	8	−1.0986	0.0000	0.0051	−1.1447	0.0001	0.0055
*Prkcd*	NM_011103	14	−1.0667	0.0000	0.0000	−1.1190	0.0001	0.0055
*Ngfr*	NM_033217	11	−1.0178	0.0000	0.0000	−1.0344	0.0001	0.0055
*Ntrk1*	NM_001033124	3	−0.9894	0.0000	0.0000	−0.9987	0.0001	0.0055
*Avpr1a*	NM_016847	10	−0.9880	0.0000	0.0001	−1.0424	0.0001	0.0055
*Gbx1*	NM_015739	5	−0.9859	0.0000	0.0004	−1.1339	0.0001	0.0055
*Col6a3*	NM_001243009	1	−0.9442	0.0001	0.0112	−1.1950	0.0001	0.0055
*Shisa6*	NM_001034874	11	−0.9164	0.0000	0.0000	−0.9462	0.0001	0.0055
*Htr1a*	NM_008308	13	−0.8765	0.0000	0.0000	−0.8989	0.0001	0.0055
*Pld5*	NM_176916	1	−0.8610	0.0000	0.0000	−0.9883	0.0004	0.0307
*Ctxn3*	NM_001134697	18	−0.8473	0.0000	0.0000	−0.9263	0.0001	0.0055
*Pde3a*	NM_018779	6	−0.8090	0.0001	0.0108	−0.8426	0.0001	0.0055
*Zic1*	NM_009573	9	−0.7906	0.0000	0.0001	−0.8006	0.0001	0.0055
*Chat*	NM_009891	14	−0.7488	0.0000	0.0002	−0.7671	0.0001	0.0055
*Sp9*	NM_001005343	2	−0.7181	0.0001	0.0142	−0.7211	0.0001	0.0055
*Nog*	NM_008711	11	−0.6856	0.0000	0.0055	−0.6826	0.0001	0.0097
*Hs3st2*	NM_001081327	7	−0.6508	0.0003	0.0391	−0.6381	0.0001	0.0055
*Ntn4*	NM_021320	10	−0.6440	0.0001	0.0134	−0.6611	0.0001	0.0097
*Synm*	NM_207663	7	−0.6170	0.0000	0.0001	−0.7814	0.0002	0.0138
*Trpc4*	NM_001253682	3	−0.6157	0.0000	0.0103	−1.9274	0.0003	0.0243
*Slc10a4*	NM_173403	5	−0.6138	0.0001	0.0167	−0.6536	0.0001	0.0055
*Zic4*	NM_009576	9	−0.6017	0.0000	0.0007	−0.6062	0.0001	0.0055
*Elfn1*	NM_175522	5	−0.5569	0.0000	0.0045	−0.5598	0.0001	0.0055
*Slc5a7*	NM_022025	17	−0.5469	0.0000	0.0030	−0.5656	0.0001	0.0055
*Col23a1*	NM_153393	11	−0.5445	0.0000	0.0001	−0.5308	0.0001	0.0055
*Lrrc55*	NM_001033346	2	−0.5366	0.0002	0.0299	−0.5715	0.0001	0.0055
*Tmem178*	NM_026516	17	−0.5249	0.0000	0.0054	−0.5279	0.0001	0.0055
*Dgkg*	NM_138650	16	−0.5125	0.0001	0.0113	−0.5270	0.0001	0.0055
*Stx1a*	NM_016801	5	0.5159	0.0000	0.0010	0.5597	0.0001	0.0055
*L3mbtl1*	NM_001081338	2	0.5599	0.0001	0.0134	0.5238	0.0002	0.0175
*Sv2b*	NM_001109753	7	0.6092	0.0000	0.0000	0.7609	0.0006	0.0389
*Tmem215*	NM_001166009	4	0.6126	0.0003	0.0379	0.6488	0.0001	0.0055
*Hrk*	NM_007545	5	0.6365	0.0001	0.0127	0.6613	0.0001	0.0055
*Calb1*	NM_009788	4	0.6679	0.0000	0.0000	0.6607	0.0001	0.0055
*C1ql3*	NM_153155	2	0.6905	0.0003	0.0467	0.7652	0.0001	0.0055
*Bhlhe22*	NM_021560	3	0.6993	0.0001	0.0119	0.7375	0.0001	0.0055
*Coch*	NM_001198835	12	0.7013	0.0000	0.0003	0.7497	0.0001	0.0055
*Nr2f1*	NM_010151	13	0.7079	0.0000	0.0010	0.7103	0.0001	0.0055
*Nptx2*	NM_016789	5	0.7789	0.0000	0.0010	0.8747	0.0001	0.0055
*Unc13c*	NM_001081153	9	0.7824	0.0000	0.0001	0.7985	0.0001	0.0055
*Cyp26b1*	NM_001177713	6	0.8214	0.0000	0.0023	0.8481	0.0001	0.0055
*Exph5*	NM_176846	9	0.8528	0.0003	0.0393	0.8928	0.0001	0.0055
*Tcf7l2*	NM_001142924	19	0.8720	0.0000	0.0001	1.3745	0.0001	0.0055
*Frem3*	NM_001167898	8	0.8789	0.0000	0.0000	0.9520	0.0001	0.0055
*Prdm8*	NM_029947	5	1.2730	0.0001	0.0109	1.6396	0.0001	0.0055
*Slc30a3*	NM_011773	5	1.3293	0.0000	0.0000	1.3863	0.0001	0.0055
*Cyp19a1*	NM_007810	9	1.3506	0.0000	0.0045	1.6930	0.0001	0.0055
*F5*	NM_007976	1	1.5600	0.0000	0.0010	1.9068	0.0001	0.0055
*Shox2*	NM_001302358	3	1.5631	0.0001	0.0218	3.4934	0.0001	0.0055
*Otp*	NM_011021	13	1.6254	0.0000	0.0086	2.7132	0.0003	0.0243
*Neurod6*	NM_009717	6	1.7555	0.0000	0.0030	3.1023	0.0008	0.0494
*Satb2*	NM_139146	1	2.0837	0.0000	0.0000	2.4912	0.0001	0.0055
*Neurod2*	NM_010895	11	2.1360	0.0000	0.0001	3.8885	0.0003	0.0212
*Sim1*	NM_011376	10	2.2845	0.0000	0.0000	2.8305	0.0001	0.0055
*Capn11*	NM_001013767	17	2.3889	0.0000	0.0000	2.7544	0.0001	0.0055

Log fold change and adjusted-*p* or q values for the 63 differentially expressed genes in the F1 generation from the STAR/featureCounts/DESeq2 and the Galaxy TopHat/Cuffdiff RNA-Seq workflows. Genes shown in both cases are those with adjusted *p* or q values <0.05 and log fold change absolute values >0.5. DESeq2 displays the “shrunken” lfc value that accounts for high dispersion across small replicate numbers with low reads, and Galaxy Cuffdiff displays the lfc of the fragments per kilobase of transcript per million mapped reads (FPKM). The DESeq2 padj is the Benjamini-Hochberg FDR-corrected *p* value for multiple tests, and the Cuffidff q value for each gene is the Storey minimum unbiased FDR estimate for that gene.

**Table 2:** Primer sequences for qPCR.

Gene Name	Forward Primer	Reverse Primer
*Stx1a*	CCGAACCCCGATGAGAAGAC	TGCTCTTTAGCTTGGAGCGA
*NeuroD6*	ATGCGACACTCAGCCTGAAA	CTGGGATTCGGGCATTACGA
*NeuroD2*	AAGCCAGTGTCTCTTCGTGG	TTGGACAGCTTCTGCGTCTT
*Htr1a*	TACTCCACTTTCGGCGCTTT	GGAGGTAGCTCCTGATTCGC
*Calb1*	ATTTCGACGCTGACGGAAGT	TGGGTAAGACGTGAGCCAAC
*Cyp26b1*	CTGGTTGCTACAGGGTTCCG	CTCAAGTGCCTCATGGCTGA
*Cplx1*	TTGGAAGGCAGAAGACCCTGA	TTATACCCTGCCGCATGACC
*Syt1*	CAGGCCCTTAAGGATGACGA	TCCGAGTATGGCACCTTGAA
*Snap25*	CAAGGCGAACAACTGGAACG	AGCTTGTTACAGGGACACACA
*Snap23*	AGCGGGACAGAGTATCCGTA	CCATCTCCCCATGTTGCCTT
*Stxbp1*	TCACGGATTCCACACTACGC	TCCATGAGGAATTTGGTGGGA
*Vamp2*	CTGGTGGAAAAACCTCAAGATGAT	GGGTGTTAAGGACAACCGGA

## Supplementary Materials

Supplementary Materials can be found at https://www.mdpi.com/1422-0067/21/9/3129/s1.

## Abbreviations

EDC	endocrine-disrupting compound
BPA	bisphenol A
ER	estrogen receptor
Esr1	estrogen receptor alpha
GD	gestational day
PND	post-natal day
qPCR	quantitative polymerase chain reaction
SEM	standard error of the mean
Stx1a	syntaxin 1a
