# Supplementary material for: Bisphenol A Exposure in utero Disrupts Hypothalamic Gene Expression Particularly Genes Suspected in Autism Spectrum Disorders and Neuron and Hormone Signaling
Source: Int J Mol Sci. 2020 Apr 29;21(9):3129. doi: 10.3390/ijms21093129 (PMC7246794; doi:10.3390/ijms21093129)
Supplement: Supplementary file 1 [file ijms-21-03129-s001.zip › ijms-765183-for publication-Supplemental Files/BPA F1 paper Supplemental Figures.pdf]

Supplemental Figures:

Biological Process

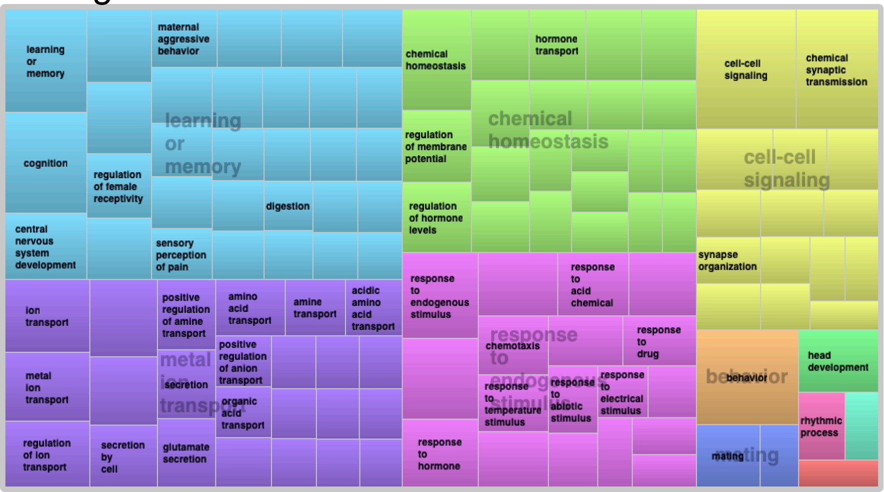

Molecular Function

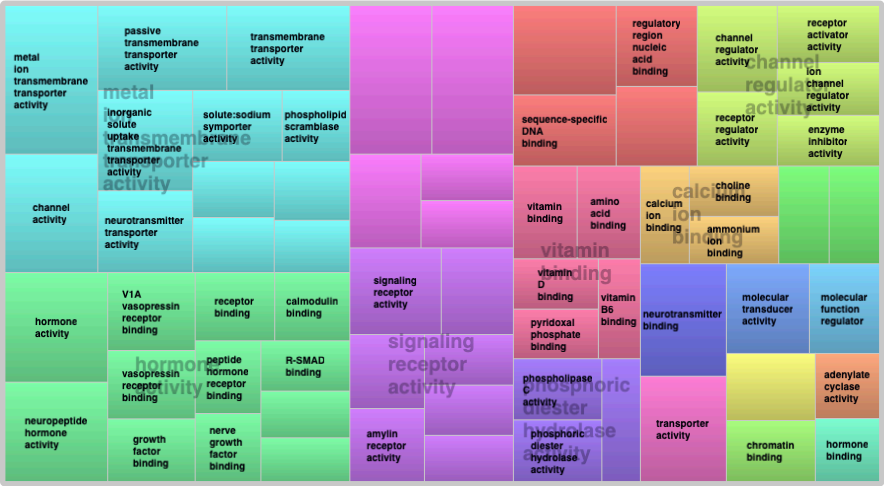

Cellular Component

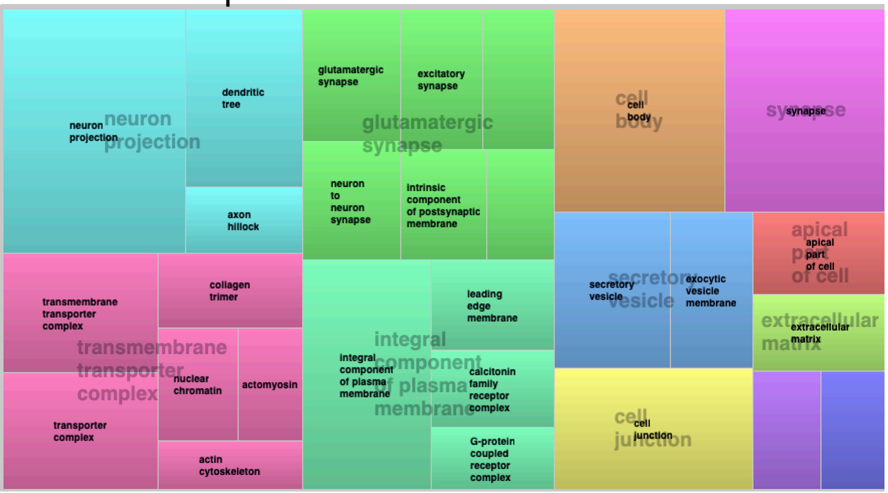

Supplemental Figure 1: REVIGO summary of Gene Ontology analysis. Significantly over-represented Molecular Functions, Biological Processes and Cellular Components were summarized using a sematic similarity algorithm.

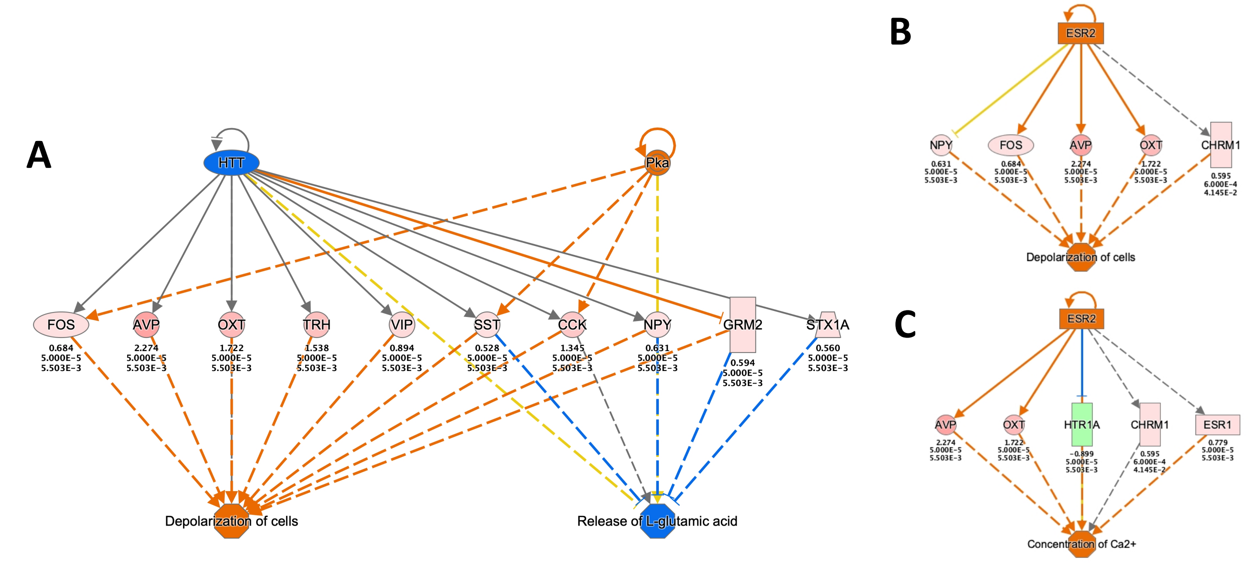

© 2000-2020 QIAGEN. All rights reserved.

Supplemental Figure 2: Ingenuity Pathway Analysis of top upstream regulators. Ingenuity generates potential hypotheses for how a function or disease is regulated in the dataset by activated or inhibited upstream regulators. Orange color indicates predicted activation. Blue color indicated predicted inhibition. Genes in pink indicate upregulation while green indicated downregulation of gene expression in BPA F1 hypothalamus. Yellow lines indicate that the findings are inconsistent with the state of the downstream molecule, while grey lines indicate the effect is not predicted.

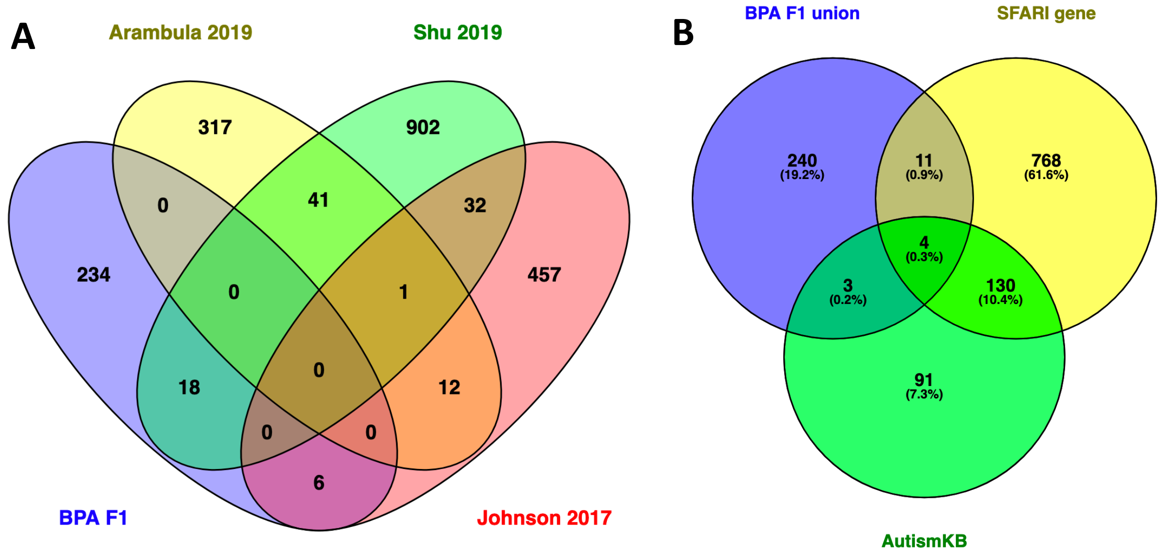

Supplemental Figure 3: Venn Diagrams of DEGs in common published datasets. A. Overlap of our DEGs (n=259 genes) with data sets published with those derived from *Mus musculus* (Shu *et al.* 2019), *Peromyscus californicus* (Johnson *et al.* 2017) and Sprague Dawley rats (Arambula *et al.* 2016); all using hypothalamus tissue. **B.** Overlap of our DEGs (n=259 genes) with two publicly available databases of genes curated from humans and animal models that are suspected to act in autism spectrum disorder: SFARI human genes and AutismKB 2.0.

References

Arambula, S. E., S. M. Belcher, A. Planchart, S. D. Turner and H. B. Patisaul (2016). "Impact of Low Dose Oral Exposure to Bisphenol A (BPA) on the Neonatal Rat Hypothalamic and Hippocampal Transcriptome: A CLARITY-BPA Consortium Study." Endocrinology **157**(10): 3856-3872. https://10.1210/en.2016-1339

Johnson, S. A., W. G. Spollen, L. K. Manshack, N. J. Bivens, S. A. Givan and C. S. Rosenfeld (2017). "Hypothalamic transcriptomic alterations in male and female California mice (Peromyscus californicus) developmentally exposed to bisphenol A or ethinyl estradiol." Physiol Rep **5**(3). https://10.14814/phy2.13133

Shu, L., Q. Meng, G. Diamante, B. Tsai, Y. W. Chen, A. Mikhail, H. Luk, B. Ritz, P. Allard and X. Yang (2019). "Prenatal Bisphenol A Exposure in Mice Induces Multitissue Multiomics Disruptions Linking to Cardiometabolic Disorders." Endocrinology **160**(2): 409-429. https://10.1210/en.2018-00817
